# Notes and News

**Published:** 1898-06-11

**Authors:** 


### Notes and News.

(Collected and Communicated.)

### HOSPITAL MEETINGS.

Hospital for Epilepsy and Paralysis.—Annual Meeting, Monday, June 13th, 3.0 p.m., at the Hospital.

Very few deaths, and only a limited number of accidents, are reported from the "road to Klondyke."

The sick and wounded from the Sierra Leone engagement are now comfortably housed in the Freetown Hospital.

A drawing-room meeting on behalf of the Invalid Children's Aid Society is to be held, by kind permission of Mrs. Taylor, at 34, Grosvenor Square, on the afternoon of June 10th.

The Goldsmiths' Company has made a grant of £600 to the building fund of the North Eastern Hospital for Children, Shoreditch, which, after remaining unfinished since 1880, will, it is hoped, soon be completed.

The entire organisation of the existing Plague Committee at Bombay has just been wound up, and the Municipal Commissioner is now in charge, and has taken over the services of the officers now on plague duty.

By the will of the late Mr. Edward Mackeson, about £10,000 is to be divided between the Royal Asylum of St. Anne's Society, the Surgical Aid Society, and the Association for Promoting the General Welfare of the Blind.

The Bristol School Board has just opened a residential home for deaf mutes, where the children will not only be taught by the lip and instructed in manual signs, but will be taught various technical occupations, whereby, it is hoped, they may become self-supporting.

It is not often that a sum so large as that of £30,000 is left to an institution which does not exist, yet this is what has practically happened in the will of the late Miss Garton, of St. Helen's, who bequeaths so generous a gift to "the St. Helen's Infirmary." There is no such institution, but there are two hospitals in St. Helen's, the one the "St. Helen's Hospital" and the other the "Providence Free Hospital," both of which institutions are naturally anxious to secure so magnificent a qequest.

"A lady," who desires to remain anonymous, has sent £1,000 to the Prince of Wales's Hospital Fund.

The Right Hon. A. J. Balfour, M.P., has consented to distribute the prizes to the successful students in the Medical School of Guy's Hospital on Wednesday, July 13th, at half-past three p.m.

The latest development of the vexed question of the site of the Newcastle Infirmary is the polling of a majority of 6,038 ratepayers' votes in favour of the Castle Leazes site. The Corporation will now go forward with a Bill to Parliament for the acquisition of this site.

A series of entertainments is to be given at the Rotunda Gardens, in Dublin, in aid of the heavy debt existing on the Rotunda Hospital. An Irish paper, speaking of the claim of this hospital to generous support, says, "In many a foreign country two facts are known about Ireland—first, that it is an island, and, second, that it contains the Rotunda Hospital." The Rotunda was the first hospital of its kind, and has done admirable work since 1745.

The Lady Elgin wards of the Lady Dufferin Hospital at Ulwar have recently been opened, and these add a new operating room and 12 rooms for *purdah* patients. During last year Miss Dissent, the medical woman in charge, and her staff, in addition to treating some 14,000 out-patients, attended upwards of 1,500 patients in their own homes; 43 major and 2,462 minor operations were performed. Miss Lander, M.D., succeeds to Miss Dissent's position.

The arrangements for the Press Bazaar in aid of the London Hospital, to be held on June 28th and 29th, are rapidly advancing. The Hospital, which has an exceptionally large space allotted to it, has already found several kind friends who have promised fruit, and it is hoped many others will send in promises during the next week. The list of stall-holders is as follows: The Lady Agnes Anderson, the Lady Muriel Watkins, the Lady Granville Gordon, the Hon. E. Kinnaird, Lady Burdett, Lady Kortright, Mrs. Skrine, Mrs. Whipple, Mrs. Woolrych Perowne, Miss Armyn Gordon, Miss Kennaway, Miss Burdett, the Misses Hare, Miss Pritchard, and Miss B. Willoughby.

An employer, writing to the *Lancet*, gives the layman's side of the question as to the proper "limits of professional secrecy," especially urging that a master's duty extends to all his servants, and that he ought to be able to ascertain what is the matter with such as are ill, so that, if the good of the rest demands it, "the peccant one" may be removed. We do not see, however, that this view of the case should in the slightest degree alter the ordinary professional rules. If a servant is ill, and the employer wishes to find out what is the matter with him, he has only to get the servant to give the medical man authority to disclose, and he will do so gladly enough. If it should be found that servants hesitate to give this authority, that would be complete proof that disclosure of such matters would be a breach of professional confidence. If, on the other hand, servants are ready to give such permission and authority, there clearly is no necessity to modify professional rules in regard to a particular class of patients. It is entirely a matter between master and servant.

